# Identifying potential inhibitors for wild-type EGFR tyrosine kinase through a cross-talk pathway strategy and an *in-silico* drug repurposing method

**DOI:** 10.1371/journal.pone.0350847

**Published:** 2026-06-22

**Authors:** Dorra Abdelmalek, Kawther Bedchich, Fahmi Smaoui, Salim Kraiem, Mohamed Ali Mosrati, Mohamed Sami Aifa

**Affiliations:** 1 Laboratory of Molecular and Cellular Screening Processes, Centre of Biotechnology of Sfax, University of Sfax, Sfax, Tunisia; 2 Department of Microbiology, Habib Bourguiba University Hospital/Faculty of Medicine of Sfax, University of Sfax, Sfax, Tunisia; Kafrelsheikh University Faculty of Pharmacy, EGYPT

## Abstract

Wild-type epidermal growth factor receptor (EGFRwt) is commonly implicated in tumor growth, yet most of the approved EGFR-targeted therapies are against mutant receptor isoforms, and patients with EGFRwt-dependent cancers have limited treatment options. We herein explored the potential to inhibit EGFRwt with five top screened hits retrieved from Food and Drug Administration (FDA)-approved kinase inhibitors library, originally developed for MET-overexpressing cancers for potential drug repurposing approach. Induced-fit docking showed that all five molecules bound the ATP-binding pocket of EGFRwt, with compound D4 emerging as the top candidate due to key interactions with ASP855 (DFG motif) and MET793 (hinge region), underscoring its favorable engagement. To further assess this interaction, we then conducted 100 ns molecular dynamics (MD) simulations, confirming the structural stability of the EGFRwt–D4 complex, with stable root-mean-square deviation (RMSD) values and maintained secondary structure elements. Free energy of binding calculations using the Molecular Mechanics/Poisson–Boltzmann Surface Area (MM/PBSA) method supported these findings, with D4 showing the most favourable interaction, dominated by Van Der Waals and electrostatic contributions from key catalytic amino acid. Our research, with further experimental validation, could be helpful to support that certain MET inhibitors, especially D4, are promising competitive inhibitors of EGFRwt, offering potential for the development of new therapeutic strategies targeting EGFRwt-driven cancers.

## Introduction

An important therapeutic challenge is the fact that most non-small cell lung cancer (NSCLC) patients are of wild-type EGFR (wtEGFR), while the majority of EGFR-directed therapies are specifically for treating activating EGFR mutations. Studies report EGFR mutations in approximately 46% to 54% of NSCLC patients depending on disease stage, ethnicity, and smoking history, particularly prevalent within adenocarcinoma subtypes and non-smokers [1,2]. Consequently, wtEGFR exists in approximately 45% to 55% of NSCLC patients, who represent a significant clinical subgroup. For instance, among patients with early-stage NSCLC, rates of mutation are 51–54%, and 46–49% are wild-type [3,4]. Similarly, among advanced NSCLC as well, frequencies of mutations of approximately 50–53% indicate that tumors fueled by wtEGFR comprise nearly half the patients [5–8].

EGFR is a transmembrane glycoprotein receptor tyrosine kinase (RTK) that is liable for guiding critical cellular activities like growth, differentiation, migration, and survival [9,10]. Ligand binding results in conformational adjustments that will allow receptor dimerization homodimerization and heterodimerization with members of the ErbB family of receptors to trigger autophosphorylation of intracellular tyrosine residues and downstream signaling cascades. These signaling cascades, MAPK/ERK, PI3K/AKT, and JAK/STAT, play critical roles in controlling cellular homeostasis and survival [9,10]. Dysregulation of these pathways, through overexpression of the receptors, has been implicated in a variety of epithelial cancers [11,12].

The last ten years have witnessed revolutionary advances with small molecule tyrosine kinase inhibitors (TKIs) addressing specifically the common EGFR mutations like deletions in exon 19 and L858R mutation in exon 21. First and second generation of TKIs like Gefitinib, Erlotinib and Afatinib have shown improved outcomes for mutation positive patients. Third-generation inhibitors, such as osimertinib, also target resistance mechanisms like the gatekeeper T790M mutation [13,14]. Targeting wtEGFR-driven cancers remains difficult in spite of these developments. Unlike mutant EGFR, which is hyperactivated, wtEGFR is tightly regulated by normal feedback mechanisms and, in the absence of overexpression, sustained activation by autocrine/paracrine ligand signaling, or defective receptor endocytosis, contributes little to oncogenesis [12,15,16].Clinically, wtEGFR overexpression correlates with poorer prognosis, reduced radiosensitivity and chemosensitivity, and increased drug resistance to EGFR-targeting agents partly due to decreased inhibitor selectivity and on-target toxicity to normal cells [7,8,17,18].

Together with EGFR, the MET receptor tyrosine kinase plays a critical role in oncogenesis, activated by hepatocyte growth factor (HGF) to regulate cell proliferation, motility, and morphogenesis. Aberrant MET activation, through overexpression or mutation, is involved in most cancers such as lung, colorectal, and breast cancer, typically associated with aggressive tumor behavior and poor clinical outcomes [19,20]. Furthermore, MET and EGFR signaling pathways have significant crosstalk, which favors compensatory mechanisms for tumor progression and resistance to monotherapy against either receptor [21–23]. Given the structural homology in the MET and EGFR kinase domains and their common roles in cancer biology, repurposing of FDA-approved MET inhibitors to target EGFRwt is a reasonable and faster therapeutic strategy, which may bypass built-in resistance and expand the treatment arsenal.

In this study, we aim to establish a rational drug repurposing framework to identify selective inhibitors targeting the wild-type epidermal growth factor receptor (wtEGFR). These inhibitors have been screened from a list of FDA-approved wild type overexpressed MET targeted TKIs. The study involves exploring the structural and energetic properties of the chosen compounds within the EGFR kinase domain through molecular docking and 100 ns molecular dynamics simulations. Additionally, binding free energies were calculated using MM/PBSA to assess the thermodynamic favorability of ligand-receptor interactions. This integrative computational method examines this set of clinically approved MET targeted TKIs as potential inhibitors for the wtEGFR kinase domain, ultimately aiding in the development of repurposed drugs for a significant untreated wt EGFR-driven cancer group of patients.

## Results

### Screening and selection of repurposed MET/EGFR inhibitors

**Fig 1:**
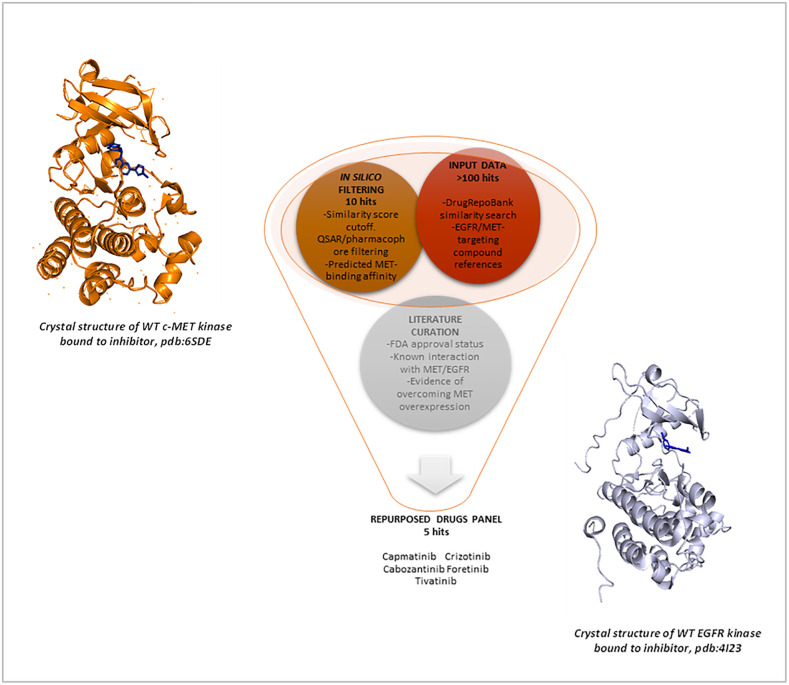
Screening chart for Identification of Repurposed MET/EGFR-Targeting Kinase Inhibitors. Schematic representation of the multi-step pipeline employed to identify FDA-approved tyrosine kinase inhibitors with potential inhibitory activity against wild-type EGFR. The process begins with similarity-based screening using DrugRepoBank and known MET/EGFR-targeting references. Subsequent in silico filtering applies pharmacophore and QSAR modeling with MET-binding affinity predictions. Final selection incorporates literature curation based on FDA approval, target engagement, and evidence of overcoming EGFR resistance. The resulting panel includes Capmatinib, Crizotinib, Cabozantinib, Foretinib, and Tivantinib.

In Fig 1, we present the process of the rationale screening and clustering of the best hits. In fact, a thorough *in silico* and literature-based screening process was employed on first set of>100 FDA-approved kinase inhibitors that could be repurposed for cancers driven by wild-type EGFR. For that, DrugRepoBank (CUHK) was used to conduct chemical similarity searches with MET and EGFR signature compounds that have been chosen on the basis of being able to bloc the overexpressed MET receptor. Compounds with the highest similarity scores were further prioritized through structure-based virtual screening methods, including QSAR modeling and pharmacophore-based filtering, focusing on the MET catalytic domain. The selection criteria were then based on predicted stable interactions with the overexpressed MET but also the potential to influence EGFR-related signaling through pathway crosstalk (10 hits at that step; yellow circle in Fig 1). Subsequently, we conducted a targeted literature review to validate the mechanistic rationale of each candidate presented in detail in Table 1, we got best 5 hits as follow: Capmatinib was approved by the FDA in May 2020 for MET exon 14, skipping NSCLC, and has demonstrated substantial antitumor efficacy in both treatment-naïve and pretreated patients. Crizotinib, a type I multi-kinase inhibitor (ALK/MET/ROS1), has shown clinical activity in MET exon 14-mutated lung cancers. Cabozantinib and Foretinib, both type II MET inhibitors, have exhibited preclinical efficacy against MET exon 14 alterations. Then, Foretinib showing superior potency against MET kinase domain in both *in vitro* and *in vivo* models. Tivantinib, a type III allosteric MET inhibitor.

**Table 1:** Pharmacological Profiles and of Top 5 Selected Repurposed MET/EGFRwt cross talk Inhibitors.

Drug	Target(s)	Pathway(s)	Key Processes Affected	EGFR Wild-Type Crosstalk
Capmatinib-D1	MET	MET/PI3K/AKT, MET/RAS/ERK	Tumor proliferation and survival	Overcomes EGFR-TKI resistance in MET-amplified NSCLC; may synergize with EGFR inhibitors in wild-type EGFR tumors.
Crizotinib-D2	ALK, ROS1, MET	ALK/PI3K/AKT/mTOR, ALK/RAS/ERK	Tumor growth, migration	Limited crosstalk with wild-type EGFR; MET inhibition may influence EGFR signaling in MET-driven cancers.
Cabozantinib-D3	MET, VEGFR2, RET, KIT	MET/PI3K/AKT, VEGFR-mediated signaling	Tumor proliferation, angiogenesis	May overcome resistance in MET-amplified cancers; multitarget activity could suppress EGFR-driven pathways in wild-type EGFR cancers.
Foretinib-D4	MET, VEGFR	MET/VEGFR-mediated pathways	Tumor growth, angiogenesis	Potential to reduce MET-dependent EGFR signaling, particularly in cancers with wild-type EGFR.
Tivantinib-D5	MET (partially), Microtubules	MET/PI3K/AKT, Cell cycle pathways	Cell survival, mitotic disruption	Limited; MET-independent effects unlikely to significantly influence wild-type EGFR signaling directly.

Therefore, as described in Table 1, these 5 drugs present an interesting serie to undertake a rigorous whole in silico study as potential EGFR kinase inhibitors repurposed from Met commercially available FDA drugs for cancer patients. This drugs set is termed as follow: Capmatinib (EGFR-D1), Crizotinib (EGFR-D2), Cabozantinib (EGFR-D3), Foretinib (EGFR-D4), and Tivantinib (EGFR-D5) and their corresponding pharmacological targets, key oncogenic pathways, and potential for crosstalk with wild-type EGFR signaling, alongside the molecular processes they influence are detailed in Table 1.

### Molecular docking analysis and interaction profiling

**Fig 2:**
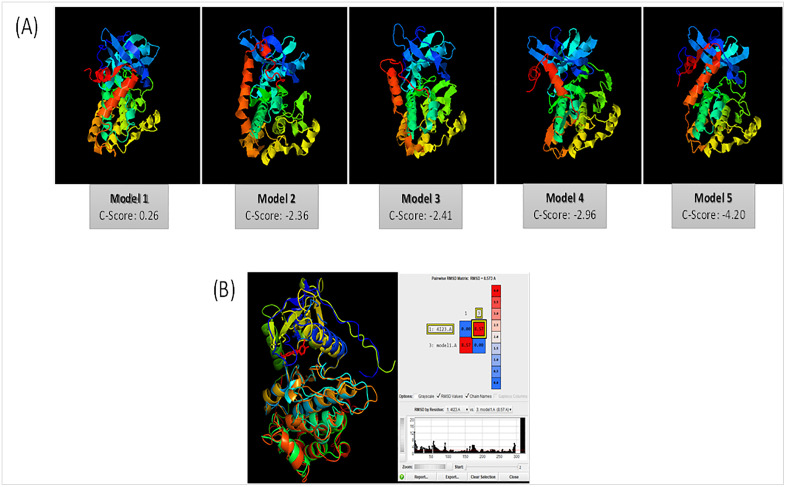
Structural Validation of EGFR Wild-Type Kinase Domain (PDB ID: 4I23) Using I-TASSER Server. Structural integrity of the EGFR wild-type kinase domain was verified using the I-TASSER server. **(A)** The top panel displays five predicted structural models generated by I-TASSER, ranked based on C-scores (confidence scores), with values ranging from –2.94 to –0.64, indicating acceptable model reliability. **(B)** The bottom panel shows a structural superposition between the experimentally resolved EGFR crystal structure (PDB ID: 4I23, in orange) and the highest-ranked I-TASSER predicted model (in green). The superimposed model confirms the absence of major secondary structure breakages. The confidence scoring plot (bottom right) and Ramachandran map further support the overall stereochemical quality of the crystal structure used in docking simulations.

We used a dual-phase molecular docking strategy to clarify the binding dynamics of repurposed MET inhibitors in the ATP-binding cleft of wild-type EGFR. Firstly, the crystal structure of EGFR (PDB ID: 4I23) was retrieved from data bank. The missing secondary structure have been cured by itasser software, as described in material and method section, in Fig 2, we show the best selected cured wt EGFR kinase model, model 1 with best confidence score (C-score) and Ramachandran plot has been retained.

AutoDock Tools 1.5–6rc3 was then used to predict binding affinities and create initial binding poses from model 1 wtEGFr kinase and the different 5 top hits. These results were then rigorously validated using the Molecular Operating Environment (MOE) software with a fitted induced docking protocol, generating 50 poses in triplicate per ligand. Double validation guaranteed the reliability and replicability of interaction patterns. All docking steps are described in M&M section and the docking poses of the ligands were evaluated using a scoring function based on binding energy. Each pose was ranked according to its binding affinity, and the top-scoring complex was selected for further analysis.

To estimate the potency of each ligand, the predicted inhibition constant (Ki) was derived from the binding free energy (ΔG) using the following equation:


$$\begin{array}{c} \text{pKi }=\;-{\text{log}}_{\text{10}}(\text{Ki}) \end{array}$$

$$\begin{array}{c} \text{Ki }=\text{ exp}(\Delta \text{G }/\;(\text{R }\times \text{ T})) \end{array}$$

Where:

- ΔG is the binding free energy in kcal·mol ^−1^
- R is the gas constant (1.98 cal·mol ^−1^·K ^−1^)
- T is the temperature in Kelvin (298.15 K)

Another key parameter used to assess ligand quality is the Ligand Efficiency (LE), which represents the binding energy per non-hydrogen atom of the ligand. It is calculated using:


$$\begin{array}{c} \text{LE }=\;\Delta \text{G}/\text{N} \end{array}$$

Where:

- LE is ligand efficiency in kcal·mol ^−1^·non-H-atom ^−1^
- ΔG is the binding free energy (kcal·mol ^−1^)
- N is the number of non-hydrogen atoms in the ligand.

Given our objective to identify best FDA-approved compounds suitable for repurposing, with a primary activity targeting the overexpressed MET receptor tyrosine kinase, this docking study aims to investigate whether such compounds could exhibit cross-reactivity or dual inhibition potential against wild-type EGFR kinase. As seen in Table 2, all the compounds tested as overexpressed MET inhibitors revealed good binding of the wild-type EGFR kinase domain with docking scores ranging from –6.65 to –8.24 kcal/mol. Foretinib (EGFR-D4) revealed the strongest binding affinity (–8.24 kcal/mol), outperforming the control ATP (–7.60 kcal/mol) and other inhibitors. Significantly, its ligand efficiency (0.37 kcal/mol/non-H atom) was also quantitatively among the highest values, suggesting Foretinib is very efficient in binding the EGFR catalytic pocket relative to its size. Capmatinib (D1) and Cabozantinib (D3) also were high-binding affinity ligands (–7.65 and –7.89 kcal/mol, respectively) with comparable ligand efficiencies. Crizotinib and Tivantinib were still desirable but relatively weaker in affinity. Interestingly, Capmatinib and Tivantinib were tied for highest predicted pKi (8.5), indicative of high competitive inhibition potential under physiologic conditions despite relatively low docking energies. Moreover, we noted that the torsional energy values were within acceptable levels for all compounds, indicating that the ligands were in energetically acceptable conformations within the EGFR binding pocket.

**Table 2:** Predicted Binding Affinities and Structural Metrics of Repurposed MET Inhibitors Docked to the EGFRwt Kinase Domain.

Complex (protein—drug)	Affinity (kcal/mol)	pKi	Ligand Efficiency (kcal/mol/non-H atom)	Torsional Energy (kcal/mol)
**EGFR^wt^-D1 (Capmatinib)**	−7.6528215	8.5	0.40	2.0
**EGFR^wt^-D2 (Crizotinib)**	−6.7576723	8.0	0.35	2.5
**EGFR^wt^-D3 (Cabozantinib)**	−7.8943138	8.1	0.38	2.5
**EGFR^wt^-D4 (Foretinib)**	−8.2448206	7.5	0.37	2.0
**EGFR^wt^-D5 (Tivantinib)**	−6.6517434	8.5	0.40	2.0
**EGFR^wt^-D6 (Reference)**	−7.6077061	6.9	0.30	1.5

**Fig 3:**
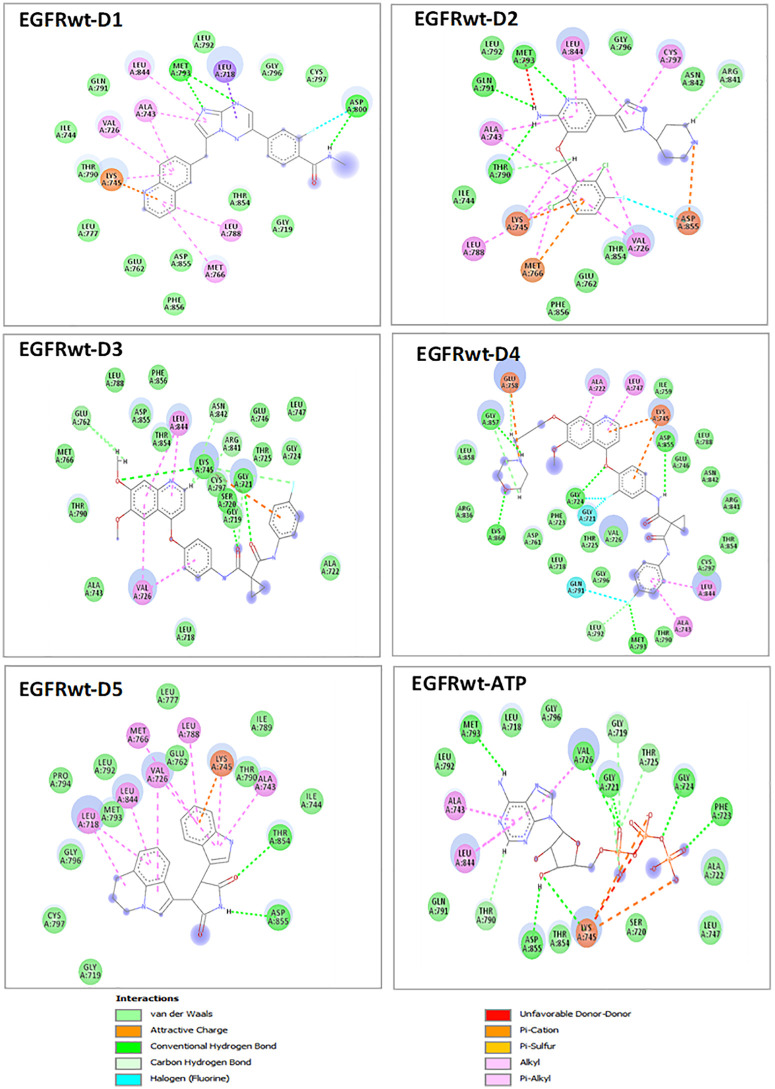
Representative 2D visualizations of the binding conformations of the five repurposed tyrosine kinase inhibitors: Capmatinib (EGFRwt-D1), Crizotinib (EGFRwt-D2), Cabozantinib (EGFRwt-D3), Foretinib (EGFRwt-D4), and Tivantinib (EGFRwt-D5) within the ATP binding pocket of wild-type EGFR. The native **ATP** molecule is included for reference to illustrate differential interaction patterns. Key residues: Gatekeeper THR790, DFG:855-857 and hinge region anchor residues: MET793 and GLN791 has been involved in different interactions such hydrogen bonding and hydrophobic interactions are annotated. Visualisation interaction analyses was performed using Discovery Studio.

As illustrated in Fig 3, the 2D interaction maps clearly demonstrate that all five ligands Capmatinib (EGFRwt-D1), Crizotinib (EGFRwt-D2), Cabozantinib (EGFRwt-D3), Foretinib (EGFRwt-D4), and Tivantinib (EGFRwt-D5) are well accommodated within the ATP-binding cleft, but differ markedly in the density and diversity of their intermolecular interactions. Foretinib (EGFRwt-D4) exhibits the most extensive interaction network, forming multiple conventional hydrogen bonds with the hinge-associated residue **GLN791** and **GLY857**, along with additional stabilization through **CYS797**. Notably, it shows strong engagement with the DFG motif through interactions with **ASP855** and **Gly857**, including π–π stacking and halogen (fluorine) contacts, indicating deep positioning within the activation loop. Additional stabilization arises from π-cation interaction with **LYS745** and π-alkyl contacts involving **LEU788**, **ALA743**, and **VAL726** (Fig 3, EGFRwt-D4).

Crizotinib (EGFRwt-D2) also demonstrates a relatively rich interaction profile, forming conventional hydrogen bonds with the hinge residue **GLN791** and **MET793**, and interacting with the DFG residue **ASP855**. It further exhibits π-cation interaction with **LYS745**, while hydrophobic stabilization is maintained through π-alkyl interactions with **LEU718**, **VAL726**, and **ALA743**. However, its interaction with the DFG motif is less extensive than that of Foretinib, particularly lacking strong engagement with **ASP855** and/or **GLY857**.

While, cabozantinib (EGFRwt-D3) presents a more compact yet efficient interaction pattern. Capmatinib (EGFRwt-D1) shows moderate interaction density, primarily through interaction with **MET793** and limited involvement of **GLN791**, whereas Tivantinib (EGFRwt-D5) displays comparatively fewer interactions, with weak hinge engagement and partial contact with the DFG region on **ASP855**. The ATP reference ligand maintains a balanced interaction network, including hydrogen bonding with hinge residues **GLN791** and **MET793**, and interactions with DFG residues **ASP855** and **GLY857**, serving as a structural and functional benchmark.

**Fig 4:**
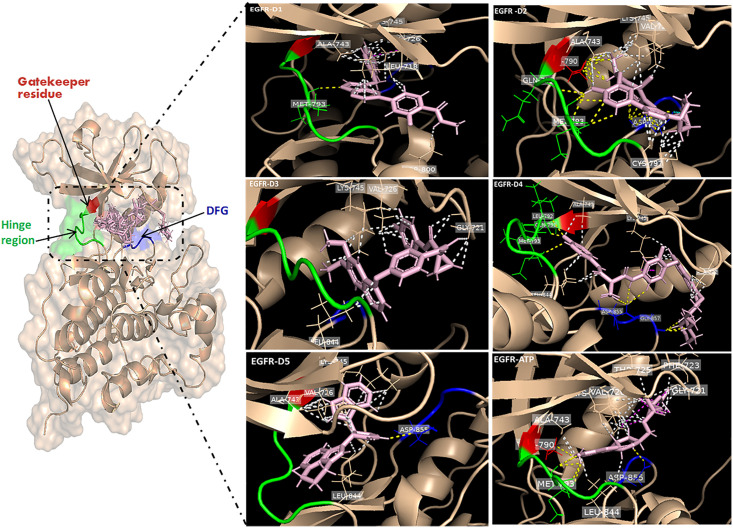
3D Binding Modes of Repurposed Drugs within the ATP-Binding Site of Wild-Type EGFR Kinase Domain. Docking poses of five repurposed kinase inhibitors: Capmatinib (EGFR-D1, red), Crizotinib (EGFR-D2, yellow), Cabozantinib (EGFR-D3, orange), Foretinib (EGFR-D4, green), and Tivantinib (EGFR-D5, cyan) within the ATP-binding pocket of the EGFR kinase domain are shown. Interacting residues forming hydrogen bonds within 3.5 Å are labeled. The left panel displays the full EGFRwt kinase, highlighting key structural regions: hinge region (green), gatekeeper THR790 (red), and DFG motif (blue). The right panel shows the superimposed binding orientations of all compounds within the pocket, with interactions involving key residues highlighted in yellow.

Visualizations of the specific intermolecular contacts formed by each ligand within the catalytic cleft are presented in Fig 4, which provides a detailed description of key hydrogen bonds, salt bridges, hydrophobic contacts, and water-mediated interactions for each complex. In fact, in Fig 4, these interaction patterns are further contextualized within the three-dimensional structure of EGFR. The right panel presents the full protein architecture, highlighting key functional regions including the ATP-binding pocket, hinge region, gatekeeper residue (THR790), and the activation loop containing the DFG motif. The left panel provides a detailed 3D view of the binding pocket, where key residues are emphasized, allowing visualization of ligand orientation and depth of insertion. Consistent with the 2D interaction analysis, Foretinib (EGFRwt-D4) demonstrates the most extensive engagement with critical residues, spanning both the hinge region (**GLN791**, **MET793**) and the DFG motif (**ASP855**, **GLY857**), confirming its optimal positioning within the active site. Crizotinib (EGFRwt-D2) follows, showing substantial with the gatekeeper **THR790** but less comprehensive interaction with these regions, while Cabozantinib (EGFRwt-D3), Capmatinib (EGFRwt-D1) and Tivantinib (EGFRwt-D5) exhibit more limited engagement. Overall, based on the qualitative interaction richness, we note that the structural insights from Fig 4 reinforce the trends observed in Fig 3, supporting the ranking of interaction strength and binding engagement as D4 > D2 > D1 > D5 > D3.

### MD simulations

The top-ranked binding pose for each protein–drug complex, as identified from the docking study, was subsequently subjected to comprehensive molecular dynamics simulations. These calculations are to examine how each compound performs under dynamic, physiologically realistic conditions so much more than static docking can tell us alone. By tracking the behavior of the complexes over 100 ns, we could observe how the ligands interacted with EGFRwt in motion, and how the protein responded accordingly and more interestingly the whole resolution of the initially retrieved protein from cristal data bank could be slightly enhanced after the MD simulations.

### Structural stability and flexibility of EGFRwt/ligand complexes

**Fig 5:**
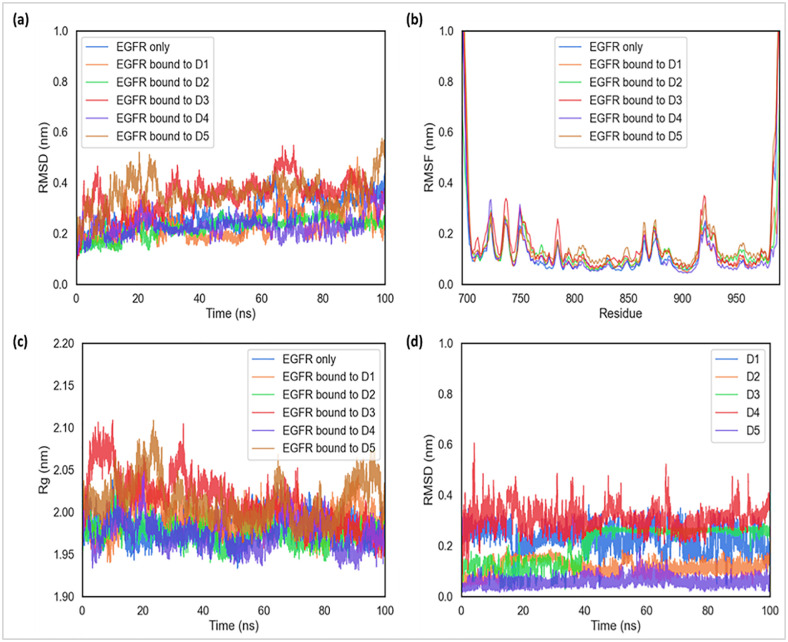
Structural stability and flexibility analysis of EGFR in complex with compounds D1, D2, D3, D4, and D5, compared to EGFR alone. **A**: Root-mean-square deviation (RMSD) of the EGFR backbone in complex with compounds D1, D2, D3, D4, and D5, compared to EGFR alone. **B**: Root-mean-square fluctuation (RMSF) of the EGFR backbone in complex with compounds D1, D2, D3, D4, and D5, compared to EGFR alone. **C**: Radius of gyration (Rg) of the EGFR backbone in complex with compounds D1, D2, D3, D4, and D5, compared to EGFR alone. **D**: Root-mean-square deviation (RMSD) of the ligand compounds D1, D2, D3, D4, and D5 during the MD simulations.

Simulations conducted for 100 ns at 310K of these five drugs within the EGFr kinase binding site and with respect to the key indicators such as root-mean-square deviation (RMSD), root-mean-square fluctuation (RMSF), ligand RMSD, and radius of gyration (Rg) were used to measure global stability and local flexibility. Results showed us first of all that the RMSD profiles, as depicted in Fig 5A, indicated that all the ligand-bound states of EGFRwt were stable during the course of the simulation, with fluctuation between 0.2 and 0.5 nm practically the same as in the unbound protein. This indicates that the binding of the ligand was not destabilizing the kinase domain, a requirement for a good inhibitor. At the residue level, RMSF analysis (Fig 5B) was found to be not significant for changes in flexibility in all the complexes, with the average fluctuations less than 0.4 nm. This consistency suggests that none of the ligands induced unwanted local perturbation to the protein backbone, signifying that the compounds are structurally compatible with the EGFRwt binding site.

Then when we look at ligand-specific RMSD (Fig 5D), which informs us about how well every compound remained in the ATP-binding pocket, we notice that D2 and D5 showed the lowest fluctuations, remaining less than 0.2 nm throughout, which reveals strong and stable binding. D1 and D4 experienced slightly greater, but still moderate, 0.2 to 0.4 nm fluctuations showing minor conformational alterations within the pocket more commonly associated with flexible adaptive binding. Importantly, D3 exhibited low drift until approximately 40 ns before stabilizing at a higher level, implying delayed but steady repositioning. Despite these minor adjustments, D4 exhibited an excellent balance between flexibility and retention, maintaining orientation within the binding pocket with reassuring stability throughout the window of simulation. Lastly, radius of gyration (Rg) information (Fig 5C) confirmed that the global compactness of EGFRwt remained intact throughout the simulation. Rg measures for every complex were between 1.95 and 2.10 nm, and D1, D2, and D4 in particular exhibited patterns that closely mirrored that of the free protein.

Thus, out of all the studied complexes, Foretinib-D4 once more exhibited stable and low-fluctuation behavior and further proved the structural compatibility and its capacity to form a tightly packed, stable complex.

### Hydrogen bounds analysis

One of the interesting outputs of MD simulations is to quantitively explore the binding affinity of these drugs in complexe with the protein through the H-bond interaction profiles.

**Fig 6:**
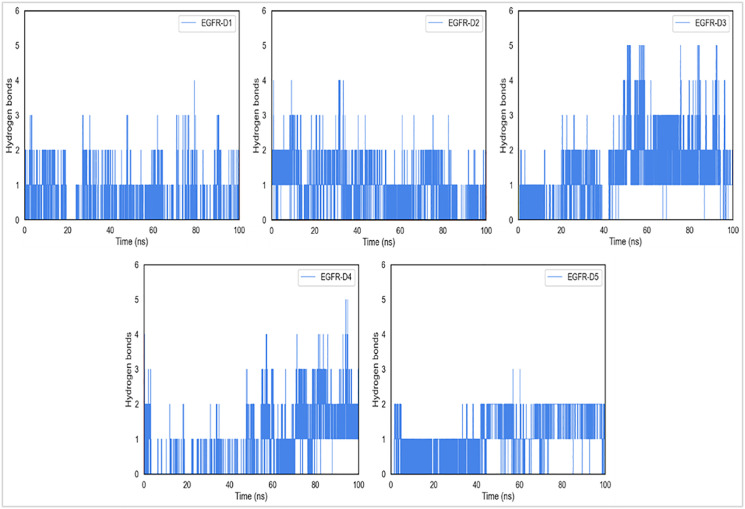
Time-Resolved Analysis of Hydrogen Bond Interactions between EGFRwt and Ligands D1–D5 during Molecular Dynamics Simulations. Hydrogen bond formation profiles of EGFRwt in complex with repurposed ligands D1 (Capmatinib), D2 (Crizotinib), D3 (Cabozantinib), D4 (Foretinib), and D5 (Tivantinib) over the course of the 100 ns molecular dynamics simulation. The number of hydrogen bonds was calculated per frame to evaluate the persistence and strength of ligand–protein interactions. Among all, EGFRwt–D4 exhibited the most consistent and sustained hydrogen bonding network, suggesting enhanced interaction stability, in agreement with docking and structural dynamics analyses.

These H-bond Interactions help to gain further insights into the nature and ligand binding stability in the EGFRwt active site. For that, we monitored the hydrogen bond formation along the 100 ns molecular dynamics trajectory. Results depicted in Fig 6 showed us that All five complexes-maintained hydrogen bonds for the duration of the simulation, although their number and stability varied among compounds.

Of note, D1 and D2 showed a similar pattern, oscillating between one and two hydrogen bonds, sometimes peaking at four. D3, however, showed a definitive increase in hydrogen bonding post the 50 ns milestone, stabilizing at two to four bonds suggesting the ligand may have been positively reshaped in the binding site. D4 (Foretinib) surprisingly showed a steep increase in hydrogen bond contacts following the simulation midpoint, culminating to five stable bonds. D5 showed enhanced bonding post 40 ns, up to as many as three stable contacts. These patterns indicate that D3, D4, and D5 could structurally modify during the course of the simulation to produce more effective, longer-lasting hydrogen bonding. Time-delayed but stabilized hydrogen bond creation points toward time-dependent accommodation, in which the ligands develop tighter, more favorable interactions with the receptor, potentially contributing to their increased binding affinity and complex stability.

### PCA projection of trajectory and free energy landscapes analysis

Finally, MD simulation informed us on the dynamical behavior of the protein-ligand complexes, via the Principal Component Analysis (PCA) and constructed Free Energy Landscapes (FEL) of the most important collective motions that we investigated also in this study. The approach provides information on how ligand binding defines the collective conformational landscape of EGFRwt and allows us to identify the most energetically favorable states visited along the course of the simulation.

**Fig 7:**
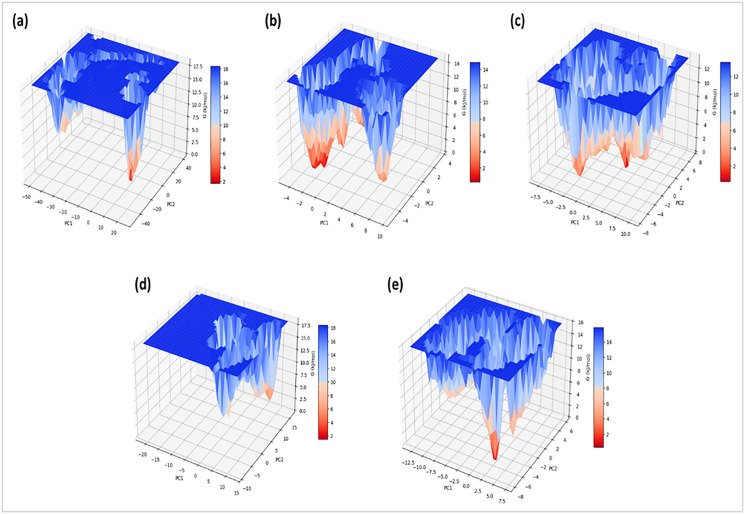
Free energy landscapes of EGFRwt complexes with D1–D5 obtained from Molecular Dynamics simulations. The plots (a–e) illustrate the Gibbs free energy surfaces projected along the first two principal components (PC1 and PC2), corresponding to the dominant motions of each complex. Distinct energy minima, visualized in red, represent stable conformational states sampled during the simulation, while blue regions indicate higher energy conformations. The number and depth of the minima reflect the conformational flexibility and binding stability of each ligand.

By projecting the trajectories onto the first two principal components, we noted the dominant motions and used these to generate FELs that highlight the basins of conformation visited by each complex (Fig 7).

Red color indicates regions of low free energy, stable conformations. For all complexes, ligand binding smoothly modified EGFRwt’s surface of energy, causing distortions in the distribution and depth of energy minima. This is an indication of a structural reorganization of the receptor in order to accommodate diverse ligands.

**Fig 8:**
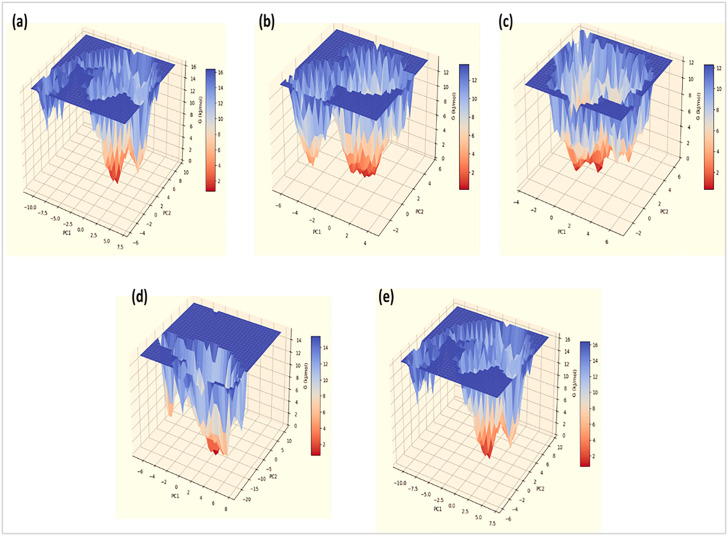
Free Energy Landscape of EGFRwt-D1-D5 Complexes During the Last 20 ns Of The Molecular Dynamic Simulation. Free energy landscapes of EGFRwt-D1 to D5 complexes during the final 20 ns of molecular dynamics simulation. The energy surfaces were constructed using principal component analysis (PCA) based on atomic fluctuations. Local minima, represented by red regions, indicate the most thermodynamically favorable conformations sampled during the equilibrium phase of each complex. Variations in the depth and distribution of energy basins reflect differences in the conformational stability and dynamic behavior of the ligand-bound EGFR-wt systems.

At 20 ns of the simulation work, the FEL profiles demonstrated that all complexes but especially and interestingly the D3, D4 and D5 hits are well stabilized within their lowest-energy states, with no indications of structural destabilization or unfolding (Fig 8).

### Binding free energy analysis (MM/PBSA)

Ultimately, to quantitatively elucidate the energetic basis of EGFRwt ligand interaction patternsfollowing their qualitative assessment through 2D and 3D visualizations (Figs 3 and 4, respectively), we performed MM/PBSA calculations. This approach enabled the decomposition of the total binding free energy into its principal contributions, including van der Waals, electrostatic, polar solvation, and non-polar solvation components (Table 3).

**Table 3:** Decomposition of Binding Free Energy for EGFRwt-Ligand Complexes.

	D1-EGFR^wt^	D2-EGFR^wt^	D3-EGFR^wt^	D4-EGFR^wt^	D5-EGFR^wt^
**ΔVDWAALS**	−18.12 ± 0.53	−31.72 ± 0.26	−28.85 ± 2.69	−37.75 ± 0.61	−34.27 ± 0.03
**ΔEEL**	−10.28 ± 1.68	28.70 ± 3.16	−27.57 ± 2.44	−26.41 ± 3.03	−25.25 ± 0.69
**ΔEGB**	20.84 ± 4.66	−9.61 ± 0.81	50.80 ± 2.36	39.88 ± 0.67	40.88 ± 0.10
**ΔESURF**	−2.19 ± 0.53	−4.17 ± 0.07	−4.16 ± 0.04	−4.87 ± 0.11	−4.39 ± 0.11
**ΔGGAS**	−28.39 ± 1.80	−3.02 ± 3.17	−56.42 ± 3.66	−64.15 ± 3.10	−59.52 ± 0.73
**ΔGSOLV**	18.65 ± 4.69	−13.78 ± 0.81	46.64 ± 2.36	35.01 ± 0.68	36.49 ± 0.15
**ΔTOTAL**	−9.74 ± 5.02	−16.79 ± 3.27	−9.78 ± 4.36	−29.15 ± 3.17	−23.03 ± 0.75

Among the five ligands, D4 (Foretinib) possessed the most favorable overall binding free energy (–29.15 ± 3.17 kcal/mol), and this was closely followed by D5 (Tivatinib, –23.03 ± 0.75 kcal/mol). The two possessed the highest affinities, which were mainly driven by strong gas-phase interactions (ΔGGAS), including van der Waals and electrostatics. Both D4 and D5 possessed other strongest ΔGGAS values (–64.15 and –59.52 kcal/mol, respectively), suggesting strong non-covalent attraction in the binding site. Van der Waals interactions played the largest role in complex stability, with D4 taking the lead at –37.75 kcal/mol, closely followed by D5 at –34.27 kcal/mol. Although electrostatic contributions varied, D2 had a highly destabilizing trend (+28.70 kcal/mol), likely due to repulsive Coulombic interactions. Solvation effects, especially polar solvation (ΔEGB), were inclined to oppose binding, with D3 being the hardest hit (+50.80 kcal/mol). On the other hand, D2 had a minor stabilizing solvation contribution (–9.61 kcal/mol). Together, these active conformations validate that D4 and D5 represent the best-suited repurposed inhibitors, in which binding is largely governed by van der Waals interactions and acceptable penalties of solvation.

## Discussion

The problem of targeting wild-type epidermal growth factor receptor (EGFRwt) in cancer therapy adequately is a recurring one. As much as mutant EGFR, i.e., that expressed in non-small cell lung cancer has driven waves of effective drug discovery, EGFRwt continues to be out of reach of effective pharmacologic manipulation. Its resistance to presently utilized EGFR inhibitors like Gefitinib, Erlotinib, and even third-generation agents like Osimertinib illustrates a therapeutic window in oncology [12,19]. But this is no minor gap: EGFRwt plays a central role in numerous aggressive cancers glioblastoma, colorectal cancer, and triple-negative breast cancer and its inhibition could radically change outcomes [20,21].

Facing these limitations, we turned to an approach that unites scientific pragmatism and rational drug discovery: drug repurposing. Rather than seeking new scaffolds de novo, we looked inward to the existing pharmacopoeia for molecules of unexploited potential. In particular, we have checked whether the FDA-approved MET inhibitors may cross the molecular chasm and bind EGFRwt, exploiting their structural relatedness and common interaction in oncogenic signaling pathways [22–24]. This is not only an efficient but also timely approach repositioning well-studied drugs can do so much to quicken clinical translation, bypassing many of the initial developmental steps.

We accessed the DrugRepoBank database to filter MET-targeting compounds for structural compatibility, pharmacokinetic favorability, and established anticancer activity. Five lead compounds: Capmatinib, Crizotinib, Cabozantinib, Foretinib, and Tivantinib, termed D1 to D5 respectively, emerged as strong contenders. Multitarget kinase profiles and well-tolerated safety profiles further enhanced their candidacy [25–27].

Molecular docking simulations provided the first insight into the binding potential of the tested compounds. All five ligands stably occupied the ATP-binding cleft of EGFRwt, recapitulating key interactions typically observed with the native ligand. Among them, Foretinib stood out not only due to its favorable binding free energy but also because of the richness of its interaction network. The overall molecular conformation of Foretinib suggested not merely a fit, but an optimal geometry tailored for effective inhibition. Other compounds showed varying degrees of engagement with critical residues reflecting moderate stabilization within the ATP-binding site. Collectively, the docking results emphasize the importance of simultaneous engagement with the hinge region (**GLN791**, **MET793**), gatekeeper (**THR790**), and DFG motif (**ASP855**, **PHE856**, **GLY857**) for effective inhibition. Among the tested compounds, Foretinib demonstrated the most extensive and favorable interactions across these key residues, supporting its potential as the lead candidate for repurposing against EGFRwt [28,29].

Then the study was strengthened by molecular dynamics (MD) simulations, which tracked how these early docking poses withstood the test of time. Foretinib excelled again with low RMSD and little fluctuation throughout the 100-ns window. This stability resolved a strong and functionally significant interaction with the kinase domain of EGFRwt. Root mean square fluctuation (RMSF) information also guaranteed that Foretinib binding reduced local residue flexibility, particularly in the activation loop-indicating a locked, inhibition-prone conformation [30,31]. Conversely, the hydrogen bonds that lasted for a long time with the hinge region suggested a stable, high-affinity interaction against displacement.

Principal Component Analysis (PCA) introduced another aspect of this tale. Foretinib-bound EGFRwt complexes, when projected onto collective motion space, demonstrated significantly smaller dynamic fluctuations compared to others a marker of conformational constraint. Free Energy Landscapes (FELs) built from these trajectories exhibited an intriguing pattern: whereas other ligands traversed wide and shallow energy basins, Foretinib plunged into a deep, sharp energetic trough, reflecting a highly favorable thermodynamic situation (Fig 7II). These FEL profiles, particularly those built using the final 20 ns of the simulation, observed the equilibrium dynamics of the complexes and confirmed the long-term conformational stability of the Foretinib–EGFRwt interaction [32,33].

To set these obtained results in terms of numbers, we turned to MM/PBSA free energy calculations. Foretinib again came out on top, with the lowest binding free energy for all energetic terms. Van der Waals and electrostatics were behind its strong binding, and modest solvation penalties ensured that these were not reversed. The overall picture was one of a ligand with both thermodynamic preference and kinetic stability a complementary and valuable combination in kinase inhibition [34–36].

The therapeutic potential of dual inhibition by Foretinib is especially amenable. Tumors will tend to become resistant by diverting their signals along different receptor tyrosine kinases. In such cases, dual-targeted treatment forms a necessity in certain patient cases, a mechanism of blocking escape pathways and imposing long-lasting responses [37–39]. Foretinib’s pharmacokinetic and safety profiles, already tested in previous-phase trials, form an imminently translatable platform into EGFRwt-driven malignancies.

Of course, these computational results, as compelling as they are, must ultimately be validated through experimental investigations. In this context, the concept of drug repurposing remains particularly significant, as it enables the rapid and cost-effective identification of new therapeutic applications for existing compounds, thereby facilitating a more efficient translation from in silico predictions to experimental and clinical validation such through biochemical kinase assays, viability assays for cancer cells, and *in vivo* models of tumors will be required to confirm Foretinib’s efficacy of inhibition and selectivity. Structural studies through crystallography or cryo-EM may even refine its manner of binding, and toxicity studies will determine its therapeutic index. If the following studies confirm our findings, Foretinib could indeed have a new purpose not as a standalone MET inhibitor, but as a dual agent that might open a blocked horizon for some anticancer treatment.

Finally, this work is more than computation. It is a call to reassess the overlooked, to repurpose the known, and to imagine alternative applications for old machines. EGFRwt, long resistant to inhibition, can still fall to molecules within our grasp.

## Conclusion

This study contributes to identify more the importance of repurposing FDA-approved MET inhibitors as anti-wild-type EGFR drugs, a receptor that is generally overlooked in present targeted therapies but is relevant in several cancers. With a robust computational method involving DrugRepoBank-based screening, molecular docking, and long molecular dynamics simulations, we identified Foretinib as a probable dual inhibitor. Among all the candidates tested, Foretinib had the most favorable binding to the EGFR ATP-pocket with a conformation close to crucial interactions of the native ligand. Its EGFRwt complex was time-stable with minimal structural fluctuations and stable hydrogen bonds and implied favorable dynamics for effective kinase inhibition. Supporting biophysical characterizations by principal component analysis and free energy landscape mapping confirmed the EGFRwt–Foretinib complex conformation stability. Additionally, MM/PBSA binding energy calculations confirmed its improved interaction profile over other screened compounds. Together, these results suggest that Foretinib not only possesses high binding affinity to EGFRwt but also benefits from a dynamic and energetic profile that supports therapeutic effectiveness. With its existing clinical history and dual MET/EGFR targeting capabilities, Foretinib could candidate for repositioning therapy for EGFRwt-driven tumors. Such a strategy would likely broaden the scope of precision oncology by offering a ready and cost-effective treatment in cancers where such targeted therapy that currently is less explored.

## Materials and methods

### Drug selection and screening strategy

To identify appropriate drug candidates for repurposing in EGFR wild-type cancers, a three-step strategy was employed, integrating computational screening with literature-based filtering. Initially, the DrugRepoBank platform (https://awi.cuhk.edu.cn/DrugRepoBank) facilitated chemical similarity searches based on established MET and EGFR pathway inhibitors. Compounds exhibiting high similarity scores underwent further evaluation through structure-based virtual screening methods, including QSAR modeling and pharmacophore alignment, to predict their affinity for the overexpressed MET kinase domain. Subsequently, a targeted literature review was conducted to assess the mechanistic relevance of the shortlisted compounds, emphasizing their documented ability to modulate MET signaling and interact with EGFR-related pathways. Only drugs with FDA approval for other cancer types and established pharmacological profiles were retained for further analysis. This integrative approach enabled the selection of five hits out of more than 100 candidates as promising candidates for repurposing in EGFR wild-type cancer models.

### Molecular docking

Molecular docking was conducted to identify potential therapeutic candidates targeting the wild-type epidermal growth factor receptor (EGFRwt). In this study targeting wild-type EGFR, the 4WRG structure (1.9 Å resolution) was initially selected; however, it exhibited significant gaps in secondary structural elements. These deficiencies were addressed using the I-TASSER (https://zhanggroup.org/I-TASSER) server to reconstruct missing regions and correct chain discontinuities. Despite this refinement, comparative analysis indicated that the 4I23 (2.8 Å resolution) structure more closely resembles the wild-type human EGFR. Moreover, as our objective is to accurately target key residues involved in competitive inhibition, 4I23 was ultimately selected as the more suitable structure for this study. This latter was prepared using AutoDock Tools 1.5–6rc3 and preparation steps involved the removal of crystallographic water molecules, the addition of polar hydrogens, and the assignment of Gasteiger partial charges to ensure accurate representation of the receptor’s electronic environment. The ligand library comprising five (n = 5 + ATP reference) FDA-approved drugs was curated to explore drug repurposing potential. Ligands were energy-minimized and converted to the appropriate file format for docking. Docking simulations were first performed using AutoDock Tools 1.5–6rc3. The workspace was defined by a grid box centered on the ATP-binding site of EGFR, with coordinates set to center_x = 1.640, center_y = 54.060, and center_z = –22.010. The grid box dimensions were 40 Å × 50 Å × 50 Å along the X, Y, and Z axes, respectively, providing comprehensive coverage of the active site and surrounding key residues.

Docking parameters included an energy range of 4 kcal/mol and an exhaustiveness of 8 to optimize the balance between accuracy and computational efficiency. Binding affinities were ranked based on predicted binding free energies (kcal/mol), and the top-ranked compounds were selected for further analysis.

To enhance the docking outcomes and simulate a scenario that more closely resembles physiological interactions, an additional round of induced-fit docking was performed using the Molecular Operating Environment (MOE 2024.06) software. Each ligand was docked into the ATP-binding site of the wild-type EGFR while ATP was present to consider competitive binding. For every compound, 50 poses were generated three times, allowing for the conformational flexibility of both the ligand and the protein side chains. This induced-fit docking approach facilitated a more precise evaluation of binding competitiveness and structural accommodation within the active site.

Optimal binding poses were visualized using BIOVIA Discovery Studio Visualizer (discovery-studio-visualizer 2025) to characterize key molecular interactions, including hydrogen bonding, hydrophobic contacts, and π-π stacking, between the ligands and EGFR active site residues.

### Molecular dynamics simulations

To assess the stability and dynamic behavior of the protein-ligand complexes, molecular dynamics (MD) simulations were performed using GROMACS 2020 with the CHARMM36 force field for protein parameterization and the Swissparam for ligand topology generation. The complexes were solvated in a TIP3P water model within a cubic box, and Na + /Cl− ions were added to neutralize the system. Energy minimization was conducted using the steepest descent algorithm to remove steric clashes and optimize the system. Equilibration was performed in two phases: constant volume and temperature (NVT) ensemble for 100 ps, followed by continuous pressure and temperature (NPT) ensemble for another 100 ps, using the V-rescale thermostat and Parrinello-Rahman barostat, respectively, to maintain physiological conditions. MD simulations were run for 100 ns at 310 K to evaluate ligand stability within the EGFR binding site. Post-simulation analyses included root mean square deviation (RMSD), root mean square fluctuation (RMSF), radius of gyration (Rg), hydrogen bonding interactions, principal component analysis (PCA), and free energy landscape (FEL) estimations.

### Binding free energy calculations

Post-MD simulations, binding free energy calculations were carried out to quantitatively evaluate the binding affinities of the chosen inhibitors and to supplement the docking outcomes. These calculations utilized the molecular mechanics Poisson–Boltzmann surface area (MM/PBSA) and molecular mechanics generalized Born surface area (MM/GBSA) methods, executed through the gmx_mmpbsa package. To ensure statistical reliability, 100 evenly distributed snapshots were taken from the last 20 ns of molecular dynamics (MD) simulation trajectories. This sampling period was selected to reflect the equilibrated state of the protein-ligand complexes. The decomposition of binding free energy included essential energetic components: van der Waals interactions, electrostatic interactions, polar solvation energy, and non-polar solvation energy. These detailed energetic profiles allowed for the analysis of the thermodynamic contributions influencing ligand binding, offering crucial insights into the molecular factors that determine complex stability.

### Statistical analysis

Statistical analysis was conducted on all quantitative data derived from docking scores, molecular dynamics simulations, and binding free energy analyses using a one-way analysis of variance (ANOVA). To determine statistically significant differences among group means, Duncan’s multiple range test was employed for multiple comparisons. A p-value of less than 0.05 was considered significant. All statistical analyses were performed using SPSS software, version 17.0 for Windows.
