# Supplementary material for: Identifying potential inhibitors for wild-type EGFR tyrosine kinase through a cross-talk pathway strategy and an in-silico drug repurposing method
Source: PLoS One. 2026 Jun 22;21(6):e0350847. doi: 10.1371/journal.pone.0350847 (PMC13286212; doi:10.1371/journal.pone.0350847)
Supplement: Attachment — Submitted filename: respond to reviewers.docx [file pone.0350847.s002.docx]

***Response to Decision Letter and Reviewer Comments***

***(Manuscript number:*** PONE-D-26-01840)

Manuscript title: Identifying Potential Inhibitors for Wild-Type EGFR Tyrosine Kinase through a Cross-Talk Pathway Strategy and an In-Silico Drug Repurposing Method

Dear Respected Professor Wagdy M. Eldehna,

Academic Editor

Journal of Plos one

March, 25, 2026

We greatly appreciate your kind request to revise our manuscript in the light of those constructive and insightful peer review comments. We are thankful for showing interest in our work. We have taken into consideration all suggestions and comments and have critically edited and revised the manuscript accordingly to comply with all suggestions. Also a thorough revision of the whole manuscript has been conducted.

We would therefore be pleased to resubmit a revised version of our improved manuscript which addresses all concerns and requests. Those changes are highlighted within the manuscript in yellow.

Please find below our response, for every single point raised by the reviewers. All page numbers refer to the final revised manuscript file with track changes in yellow.

We would be glad if you could consider our revised manuscript for possible publication in the Journal of Plos one

Once again, thank you indeed for your kind consideration.

On behalf of all co-authors,

Corresponding author:
Dr. Dorra Abdelmalek
Laboratory of Molecular and Cellular Screening Processes,

Centre of Biotechnology of Sfax,

University of Sfax, 3041 Sfax, Tunisia
Email: dorra.abdelmalek@cbs.rnrt.tn

**Reviewer #1**: Dear Author,

Thank you for your submission.
While the docking study is promising, several methodological concerns must be addressed to strengthen the manuscript.

**R1**.Protein Structure Selection
The chosen EGFR kinase domain structure (PDB ID: 4I23) has a resolution of 2.80 Å, which is suboptimal for precise docking studies due to potential inaccuracies in atomic coordinates, particularly in the ATP-binding pocket. Higher-resolution structures (<2.0 Å) or those with improved electron density for key residues should be prioritized.

**A1**. We sincerely appreciate your insightful and constructive comment. Prior to selecting the protein structure that serves as the foundation of this study, a thorough and rigorous evaluation of available database entries was conducted to ensure suitability. We fully understand that high resolution structures are strongly recommended for molecular docking and molecular dynamics simulations. In our investigation, which specifically targets the wild-type epidermal growth factor receptor (EGFR) in its crystallographic form, we initially considered the 4WRG structure, characterized by a resolution of 1.9 Å. However, this structure exhibited substantial deficiencies in secondary structural elements, including α-helices, β-sheets, and loop regions.

To address these limitations, the 4WRG structure was refined using the I-TASSER server (<https://zhanggroup.org/I-TASSER>) to reconstruct missing secondary structures and resolve chain discontinuities. Nevertheless, upon comparative analysis with the 4I23 structure (afetr being cured with I-tasser), we determined that the latter exhibits a higher degree of structural similarity to the wild-type human EGFR, thereby making it more appropriate for our study. There are also other strcuture but with resolution >2A like cited in the table below :

| PDB ID | Resolution | Notes |
| --- | --- | --- |
| 4WRG | **1.90 Å** | Wild-type kinase domain |
| 9BY4 | 2.31 Å | WT EGFR + non-covalent osimertinib |
| 9BY6 | 2.55 Å | WT EGFR soaked with inhibitor |
| 2GS2 | 2.80 Å | Active EGFR kinase domain |

Taking this concern into account, as kindly highlighted, we have addressed and clarified this point in the revised manuscript Molecular Docking M&M section, **page 17-18, lines 550-557**.

Furthermore, as the present work primarily relies on molecular dynamics simulations and related calculations that require high structural reliability, the selected 4I23 structure demonstrated notable stability and yielded meaningful results throughout the study. As known, molecular dynamics simulations performed using GROMACS contributed to a slight improvement in structural refinement. The docking analysis was subsequently complemented by a rigorous dynamic evaluation of both the backbone protein and its ligand-bound complexes. Key parameters including root-mean-square deviation (RMSD), root-mean-square fluctuation (RMSF), ligand RMSD, and radius of gyration (Rg) along with Principal Component Analysis (PCA) and Free Energy Landscape (FEL) construction, provided detailed insights into the protein’s dynamical behavior. These additions have been incorporated into the main manuscript **page 10, lines 289–293**.

**R2**.Target Pocket Visualization
Clearer images of the target binding pocket are needed, explicitly labeling essential amino acids (e.g., hinge region residues, gatekeeper, DFG motif). Current figures lack sufficient detail for readers to assess interaction validity.

**A2**. We appreciate the reviewer’s valuable comment regarding the need for clearer visualization of the target binding pocket and explicit labeling of essential amino acids. In response, Figures 3 hasbeen thoroughly revised to improve clarity, structural interpretation, and interaction transparency as we re-undertook more induced-fit docking protocol (100 runs) to refine ligand conformations within the binding pocket and ensure robustness of the observed interactions. The revised Figure 3 (2D interaction maps) now provides detailed residue-level interactions, clearly showing hydrogen bonds, π-cation, π–π stacking, and hydrophobic contacts involving the key residues mentioned above. Notably, strong and recurrent interactions with **ASP855** (DFG motif), including electrostatic and π-related contacts, were observed for the top-performing compounds.

Specifically, we have refocused the analysis on key functional residues within the EGFR active site, including the hinge region (**GLN791**, **MET793**), the gatekeeper residue (**THR790**), and the DFG motif (**ASP855**, **PHE856**, **GLY857**). These residues are now explicitly highlighted and labeled in the updated figures to allow readers to better assess ligand–protein interaction validity.

A new Figure 4 has also been introduced to provide a comprehensive structural context: the left panel displays the full EGFR protein with major functional regions annotated, while the right panel offers a detailed 3D visualization of the binding pocket with key residues highlighted. This dual representation allows for better assessment of ligand positioning relative to critical structural elements.

Please check the revised version of the manuscript on Results section **pages 8-9- lines 236-281 and discussion at page 14-15 lines 451-462.**

**R3**. Binding Mode Analysis
Notably, the docked hit compounds show no interactions with critical ATP-site residues MET793 or GLN791, which are essential for EGFR inhibitor stability (as seen in co-crystallized dacomitinib). This absence undermines claims of suitable binding poses and compound efficacy.

**A3.** We thank the reviewer for this important and insightful observation regarding the absence of interactions with key hinge residues **MET793** and **GLN791**, which are indeed critical for stabilizing ATP-competitive EGFR inhibitors. We acknowledge as previously described that the initial interaction plots did not adequately capture these contacts, primarily due to the specific docking poses selected for visualization. To address this, we have performed additional and more rigorous induced-fit docking (100 runs) as mentionned in the previous remark, allowing better sampling of ligand conformations within the ATP-binding pocket.

As a result, the revised analyses now clearly demonstrate interactions with the hinge region, particularly involving **GLN791** and **MET793**, as well as the gatekeeper residue **THR790** and the DFG motif (**ASP855**, **PHE856**, **GLY857**). These interactions are now explicitly highlighted in the updated figures.

Specifically, revised Figure 3 provides an improved 2D representation of the binding pocket, where ligand positioning relative to these key residues is clearly visible. In addition, a new Figure 4 has been included to explicitly present the 3D interaction profiles of all compounds with the critical hinge residues (**GLN791**, **MET793**) and other essential structural elements.

**Reviewer #2**: There is a jump from computational prediction to the implications of therapeutic effectiveness.

**R1**. There is no benchmarking with established EGFRwt inhibitors; therefore, the binding affinity and dynamics cannot be fully evaluated.
No comparison with known EGFR inhibitors.

**A1.** We sincerely appreciate your valuable comment regarding the importance of benchmarking against established EGFRwt inhibitors. As correctly noted, the present study is grounded in a drug repurposing strategy, transitioning from c-MET targeted compounds to EGFR, specifically the wild-type receptor. The investigated molecules were selected from a library of Food and Drug Administration (FDA)-approved kinase inhibitors.

Given the high degree of structural conservation within the kinase family, our objective was to explore the potential of these approved compounds to interact effectively with EGFRwt through rigorous in silico simulations, thereby identifying promising candidates in a time- and cost-efficient manner. We fully acknowledge the importance of benchmarking ; however, the primary scope of this work is predictive screening.

Importantly, we emphasize the critical role of subsequent bench experimental validation to confirm these findings. In this regard, we have added a dedicated paragraph in the revised manuscript (**page 16, lines 495–501**) highlighting the necessity of experimental benchmarking against established EGFR inhibitors to substantiate and extend the computational results.
