# Supplementary material for: Identifying potential inhibitors for wild-type EGFR tyrosine kinase through a cross-talk pathway strategy and an in-silico drug repurposing method
Source: PLoS One. 2026 Jun 22;21(6):e0350847. doi: 10.1371/journal.pone.0350847 (PMC13286212; doi:10.1371/journal.pone.0350847)
Supplement: Attachment — Submitted filename: Response to Reviewers.docx [file pone.0350847.s003.docx]

***Response to Decision Letter and Reviewer Comments***

***(Manuscript number:*** PONE-D-26-01840R1)

Manuscript title: Identifying Potential Inhibitors for Wild-Type EGFR Tyrosine Kinase through a Cross-Talk Pathway Strategy and an In-Silico Drug Repurposing Method

**Dear Respected Professor Wagdy M. Eldehna,**

Academic Editor

Journal of Plos one

Mai, 12, 2026

We greatly appreciate your kind appreciation of the revision made to make the paper as its best scientific level. In this minor revision, we have taken also into consideration all Highly Respectful suggestions and comments raised by the reviewer.

We would therefore be pleased to resubmit a revised version of our improved manuscript which addresses all concerns and requests. Those changes are highlighted within the manuscript in yellow.

Please find below our response, for every single point raised by the reviewer. All page numbers refer to the final revised manuscript file with track changes in yellow.

We would be glad if you could consider our revised manuscript for possible publication in the Journal of Plos one

Once again, thank you indeed for your kind consideration.

On behalf of all co-authors,

Corresponding author:
Dr. Dorra Abdelmalek
Laboratory of Molecular and Cellular Screening Processes,

Centre of Biotechnology of Sfax,

University of Sfax, 3041 Sfax, Tunisia
Email: dorra.abdelmalek@cbs.rnrt.tn

**Reviewer #3**: The revised manuscript by Abdelmalek et al. represents a meaningful response to the concerns raised in the first review cycle. The authors have addressed the two most critical methodological weaknesses: the structural justification for PDB ID 4I23 is now clearly articulated in the Materials and Methods section, and the binding mode analysis has been significantly strengthened through re-execution of induced-fit docking at 100 runs, with the revised figures now explicitly demonstrating interactions with the pharmacologically essential hinge residues GLN791 and MET793, the gatekeeper THR790, and the DFG motif. The addition of Figure 4 providing dual 2D/3D visualization of the binding pocket represents a genuine improvement in interpretive clarity. The computational pipeline, combining DrugRepoBank screening, dual-software docking validation, 100 ns GROMACS molecular dynamics, PCA/FEL analysis, and MM/PBSA free energy decomposition, is methodologically appropriate for a repurposing study of this scope, and Foretinib's emergence as the lead candidate is consistently and convergently supported across all metrics. The drug repurposing rationale exploiting MET/EGFR pathway crosstalk is scientifically well-grounded and clinically relevant given the underserved EGFRwt patient population.

However, prior to final acceptance, three minor but necessary corrections must be addressed:

**R1**. The AutoDock Tools version placeholder ("version X.X") at line 558 must be completed.

**A1**. Yes, indeed it is Autodock tools -1.5.6rc3. we corrected this in the revised manuscript at **lines 186, 554 and 559**.

**R2.** The textual claim that D4 exhibits the highest ligand efficiency should be reconciled with Table 2, which shows D1 and D5 at 0.40 versus D4 at 0.37 The ranking of interaction strength stated in the text at lines 280–281 (D4 > D2 > D1 > D5 > D3) is based on qualitative structural engagement rather than raw docking affinity scores, which follow a different order in Table 2 (D4 > D3 > D1 > D2 > D5). While this distinction is scientifically defensible, it is never explicitly acknowledged in the text and is likely to confuse readers. I believe that a brief clarifying sentence noting that the two rankings reflect different evaluation criteria, quantitative binding affinity versus qualitative interaction richness, would resolve this ambiguity.

**A2.** Thank you for this interesting remark and for the suggestion to resolve the ambiguity. For that we corrected that D ligand efficiency score is among the best quantitative score, **lines 221 and 222.**

And for the qualitative scoring order we changed the text as follow: “Overall, based on the qualitative interaction richness, we note that the structural insights from Figure 4 reinforce the trends observed in Figure 3, supporting the ranking of interaction strength and binding engagement as D4 > D2 > D1 > D5 > D3. » **lines 279-281 in the revised manuscript**.

**R3.** Additionally, despite the authors' acknowledgement of language editing support, several typographical errors persist throughout the manuscript (e.g., "describved," "strenghted," "trmed," "Simualtions," "rigourous," "biddable" and "leftt") and should be corrected before publishing the paper.

**A3**. Thank you for taking care of these typing mistakes. As per recommended all these typo errors has been carefully corrected and highlighted in yellow.
