# Supplementary material for: Identifying potential inhibitors for wild-type EGFR tyrosine kinase through a cross-talk pathway strategy and an in-silico drug repurposing method
Source: PLoS One. 2026 Jun 22;21(6):e0350847. doi: 10.1371/journal.pone.0350847 (PMC13286212; doi:10.1371/journal.pone.0350847)
Supplement: Peer review history

# Peer review: Decision Letter 0

**DOI:** 10.1371/journal.pone.0350847.r001

**Authors:** Wagdy Eldehna

3 Mar 2026

PONE-D-26-01840

Identifying Potential Inhibitors for Wild-Type EGFR Tyrosine Kinase through a Cross-Talk Pathway Strategy and an In-Silico Drug Repurposing Method

PLOS One

Dear Dr. Abdelmalek,

Thank you for submitting your manuscript to PLOS ONE. After careful consideration, we feel that it has merit but does not fully meet PLOS ONE’s publication criteria as it currently stands. Therefore, we invite you to submit a revised version of the manuscript that addresses the points raised during the review process.

Please submit your revised manuscript by Apr 17 2026 11:59PM. If you will need more time than this to complete your revisions, please reply to this message or contact the journal office at plosone@plos.org. When you're ready to submit your revision, log on to https://www.editorialmanager.com/pone/ and select the 'Submissions Needing Revision' folder to locate your manuscript file.

Please include the following items when submitting your revised manuscript:

- A letter that responds to each point raised by the academic editor and reviewer(s). You should upload this letter as a separate file labeled 'Response to Reviewers'.
- A marked-up copy of your manuscript that highlights changes made to the original version. You should upload this as a separate file labeled 'Revised Manuscript with Track Changes'.
- An unmarked version of your revised paper without tracked changes. You should upload this as a separate file labeled 'Manuscript'.

If you would like to make changes to your financial disclosure, please include your updated statement in your cover letter. Guidelines for resubmitting your figure files are available below the reviewer comments at the end of this letter.

If applicable, we recommend that you deposit your laboratory protocols in protocols.io to enhance the reproducibility of your results. Protocols.io assigns your protocol its own identifier (DOI) so that it can be cited independently in the future. For instructions see: https://journals.plos.org/plosone/s/submission-guidelines#loc-laboratory-protocols. Additionally, PLOS ONE offers an option for publishing peer-reviewed Lab Protocol articles, which describe protocols hosted on protocols.io. Read more information on sharing protocols at https://plos.org/protocols?utm_medium=editorial-email&utm_source=authorletters&utm_campaign=protocols.

We look forward to receiving your revised manuscript.

Kind regards,

Wagdy M. Eldehna, Ph.d

Academic Editor

PLOS One

Journal requirements:

When submitting your revision, we need you to address these additional requirements.

1. Please ensure that your manuscript meets PLOS ONE's style requirements, including those for file naming. The PLOS ONE style templates can be found at

https://journals.plos.org/plosone/s/file?id=wjVg/PLOSOne_formatting_sample_main_body.pdf and

https://journals.plos.org/plosone/s/file?id=ba62/PLOSOne_formatting_sample_title_authors_affiliations.pdf

2. Please note that PLOS One has specific guidelines on code sharing for submissions in which author-generated code underpins the findings in the manuscript. In these cases, we expect all author-generated code to be made available without restrictions upon publication of the work. Please review our guidelines at https://journals.plos.org/plosone/s/materials-and-software-sharing#loc-sharing-code and ensure that your code is shared in a way that follows best practice and facilitates reproducibility and reuse.

3. Please include captions for your Supporting Information files at the end of your manuscript, and update any in-text citations to match accordingly. Please see our Supporting Information guidelines for more information: http://journals.plos.org/plosone/s/supporting-information.

If the reviewer comments include a recommendation to cite specific previously published works, please review and evaluate these publications to determine whether they are relevant and should be cited. There is no requirement to cite these works unless the editor has indicated otherwise.

[Note: HTML markup is below. Please do not edit.]

Reviewers' comments:

Reviewer's Responses to Questions

**Comments to the Author**

1. Is the manuscript technically sound, and do the data support the conclusions?

The manuscript must describe a technically sound piece of scientific research with data that supports the conclusions. Experiments must have been conducted rigorously, with appropriate controls, replication, and sample sizes. The conclusions must be drawn appropriately based on the data presented.

Reviewer #1: Yes

Reviewer #2: Partly

**********

2. Has the statistical analysis been performed appropriately and rigorously?

Reviewer #1: N/A

Reviewer #2: N/A

**********

3. Have the authors made all data underlying the findings in their manuscript fully available?

The PLOS Data policy requires authors to make all data underlying the findings described in their manuscript fully available without restriction, with rare exception (please refer to the Data Availability Statement in the manuscript PDF file). The data should be provided as part of the manuscript or its supporting information, or deposited to a public repository. For example, in addition to summary statistics, the data points behind means, medians and variance measures should be available. If there are restrictions on publicly sharing data—e.g. participant privacy or use of data from a third party—those must be specified.

Reviewer #1: No

Reviewer #2: No

**********

4. Is the manuscript presented in an intelligible fashion and written in standard English?

PLOS ONE does not copyedit accepted manuscripts, so the language in submitted articles must be clear, correct, and unambiguous. Any typographical or grammatical errors should be corrected at revision, so please note any specific errors here.

Reviewer #1: Yes

Reviewer #2: Yes

**********

5. Review Comments to the Author

Please use the space provided to explain your answers to the questions above. You may also include additional comments for the author, including concerns about dual publication, research ethics, or publication ethics. (Please upload your review as an attachment if it exceeds 20,000 characters)

Reviewer #1: Dear Author,

Thank you for your submission.

While the docking study is promising, several methodological concerns must be addressed to strengthen the manuscript.

Protein Structure Selection

The chosen EGFR kinase domain structure (PDB ID: 4I23) has a resolution of 2.80 Å, which is suboptimal for precise docking studies due to potential inaccuracies in atomic coordinates, particularly in the ATP-binding pocket. Higher-resolution structures (<2.0 Å) or those with improved electron density for key residues should be prioritized.

Target Pocket Visualization

Clearer images of the target binding pocket are needed, explicitly labeling essential amino acids (e.g., hinge region residues, gatekeeper, DFG motif). Current figures lack sufficient detail for readers to assess interaction validity.

Binding Mode Analysis

Notably, the docked hit compounds show no interactions with critical ATP-site residues MET793 or GLN791, which are essential for EGFR inhibitor stability (as seen in co-crystallized dacomitinib). This absence undermines claims of suitable binding poses and compound efficacy.

Recommendations

Replace 4I23 with a higher-resolution EGFR structure .

Add annotated 2D interaction diagrams and 3D pocket views highlighting all key contacts.

Revise docking validation to confirm hinge hydrogen bonds and hydrophobic packing with MET793/GLN791.

Reviewer #2: There is a jump from computational prediction to the implications of therapeutic effectiveness.

There is no benchmarking with established EGFRwt inhibitors; therefore, the binding affinity and dynamics cannot be fully evaluated.

No comparison with known EGFR inhibitors

**********

6. PLOS authors have the option to publish the peer review history of their article (what does this mean?). If published, this will include your full peer review and any attached files.

If you choose “no”, your identity will remain anonymous but your review may still be made public.

**Do you want your identity to be public for this peer review?**  For information about this choice, including consent withdrawal, please see our Privacy Policy.

Reviewer #1: **Yes:**  Abdulrahman M. Saleh

Reviewer #2: **Yes:**  Mahmoud Rashed

**********

[NOTE: If reviewer comments were submitted as an attachment file, they will be attached to this email and accessible via the submission site. Please log into your account, locate the manuscript record, and check for the action link "View Attachments". If this link does not appear, there are no attachment files.]

To ensure your figures meet our technical requirements, please review our figure guidelines: https://journals.plos.org/plosone/s/figures

You may also use PLOS’s free figure tool, NAAS, to help you prepare publication quality figures: https://journals.plos.org/plosone/s/figures#loc-tools-for-figure-preparation.

NAAS will assess whether your figures meet our technical requirements by comparing each figure against our figure specifications.

# Author comment: Author response to Decision Letter 1

**DOI:** 10.1371/journal.pone.0350847.r002

25 Mar 2026

Response to Decision Letter and Reviewer Comments

(Manuscript number: PONE-D-26-01840)

Manuscript title: Identifying Potential Inhibitors for Wild-Type EGFR Tyrosine Kinase through a Cross-Talk Pathway Strategy and an In-Silico Drug Repurposing Method

Dear Respected Professor Wagdy M. Eldehna,

Academic Editor

Journal of Plos one

March, 25, 2026

We greatly appreciate your kind request to revise our manuscript in the light of those constructive and insightful peer review comments. We are thankful for showing interest in our work. We have taken into consideration all suggestions and comments and have critically edited and revised the manuscript accordingly to comply with all suggestions. Also a thorough revision of the whole manuscript has been conducted.

We would therefore be pleased to resubmit a revised version of our improved manuscript which addresses all concerns and requests. Those changes are highlighted within the manuscript in yellow.

Please find below our response, for every single point raised by the reviewers. All page numbers refer to the final revised manuscript file with track changes in yellow.

We would be glad if you could consider our revised manuscript for possible publication in the Journal of Plos one

Once again, thank you indeed for your kind consideration.

On behalf of all co-authors,

Corresponding author:

Dr. Dorra Abdelmalek

Laboratory of Molecular and Cellular Screening Processes,

Centre of Biotechnology of Sfax,

University of Sfax, 3041 Sfax, Tunisia

Email: dorra.abdelmalek@cbs.rnrt.tn

Reviewer #1: Dear Author,

Thank you for your submission.

While the docking study is promising, several methodological concerns must be addressed to strengthen the manuscript.

R1.Protein Structure Selection

The chosen EGFR kinase domain structure (PDB ID: 4I23) has a resolution of 2.80 Å, which is suboptimal for precise docking studies due to potential inaccuracies in atomic coordinates, particularly in the ATP-binding pocket. Higher-resolution structures (<2.0 Å) or those with improved electron density for key residues should be prioritized.

A1. We sincerely appreciate your insightful and constructive comment. Prior to selecting the protein structure that serves as the foundation of this study, a thorough and rigorous evaluation of available database entries was conducted to ensure suitability. We fully understand that high resolution structures are strongly recommended for molecular docking and molecular dynamics simulations. In our investigation, which specifically targets the wild-type epidermal growth factor receptor (EGFR) in its crystallographic form, we initially considered the 4WRG structure, characterized by a resolution of 1.9 Å. However, this structure exhibited substantial deficiencies in secondary structural elements, including α-helices, β-sheets, and loop regions.

To address these limitations, the 4WRG structure was refined using the I-TASSER server (https://zhanggroup.org/I-TASSER) to reconstruct missing secondary structures and resolve chain discontinuities. Nevertheless, upon comparative analysis with the 4I23 structure (afetr being cured with I-tasser), we determined that the latter exhibits a higher degree of structural similarity to the wild-type human EGFR, thereby making it more appropriate for our study. There are also other strcuture but with resolution >2A like cited in the table below :

PDB ID Resolution Notes

4WRG 1.90 Å Wild-type kinase domain

9BY4 2.31 Å WT EGFR + non-covalent osimertinib

9BY6 2.55 Å WT EGFR soaked with inhibitor

2GS2 2.80 Å Active EGFR kinase domain

Taking this concern into account, as kindly highlighted, we have addressed and clarified this point in the revised manuscript Molecular Docking M&M section, page 17-18, lines 550-557.

Furthermore, as the present work primarily relies on molecular dynamics simulations and related calculations that require high structural reliability, the selected 4I23 structure demonstrated notable stability and yielded meaningful results throughout the study. As known, molecular dynamics simulations performed using GROMACS contributed to a slight improvement in structural refinement. The docking analysis was subsequently complemented by a rigorous dynamic evaluation of both the backbone protein and its ligand-bound complexes. Key parameters including root-mean-square deviation (RMSD), root-mean-square fluctuation (RMSF), ligand RMSD, and radius of gyration (Rg) along with Principal Component Analysis (PCA) and Free Energy Landscape (FEL) construction, provided detailed insights into the protein’s dynamical behavior. These additions have been incorporated into the main manuscript page 10, lines 289–293.

R2.Target Pocket Visualization

Clearer images of the target binding pocket are needed, explicitly labeling essential amino acids (e.g., hinge region residues, gatekeeper, DFG motif). Current figures lack sufficient detail for readers to assess interaction validity.

A2. We appreciate the reviewer’s valuable comment regarding the need for clearer visualization of the target binding pocket and explicit labeling of essential amino acids. In response, Figures 3 hasbeen thoroughly revised to improve clarity, structural interpretation, and interaction transparency as we re-undertook more induced-fit docking protocol (100 runs) to refine ligand conformations within the binding pocket and ensure robustness of the observed interactions. The revised Figure 3 (2D interaction maps) now provides detailed residue-level interactions, clearly showing hydrogen bonds, π-cation, π–π stacking, and hydrophobic contacts involving the key residues mentioned above. Notably, strong and recurrent interactions with ASP855 (DFG motif), including electrostatic and π-related contacts, were observed for the top-performing compounds.

Specifically, we have refocused the analysis on key functional residues within the EGFR active site, including the hinge region (GLN791, MET793), the gatekeeper residue (THR790), and the DFG motif (ASP855, PHE856, GLY857). These residues are now explicitly highlighted and labeled in the updated figures to allow readers to better assess ligand–protein interaction validity.

A new Figure 4 has also been introduced to provide a comprehensive structural context: the left panel displays the full EGFR protein with major functional regions annotated, while the right panel offers a detailed 3D visualization of the binding pocket with key residues highlighted. This dual representation allows for better assessment of ligand positioning relative to critical structural elements.

Please check the revised version of the manuscript on Results section pages 8-9- lines 236-281 and discussion at page 14-15 lines 451-462.

R3. Binding Mode Analysis

Notably, the docked hit compounds show no interactions with critical ATP-site residues MET793 or GLN791, which are essential for EGFR inhibitor stability (as seen in co-crystallized dacomitinib). This absence undermines claims of suitable binding poses and compound efficacy.

A3. We thank the reviewer for this important and insightful observation regarding the absence of interactions with key hinge residues MET793 and GLN791, which are indeed critical for stabilizing ATP-competitive EGFR inhibitors. We acknowledge as previously described that the initial interaction plots did not adequately capture these contacts, primarily due to the specific docking poses selected for visualization. To address this, we have performed additional and more rigorous induced-fit docking (100 runs) as mentionned in the previous remark, allowing better sampling of ligand conformations within the ATP-binding pocket.

As a result, the revised analyses now clearly demonstrate interactions with the hinge region, particularly involving GLN791 and MET793, as well as the gatekeeper residue THR790 and the DFG motif (ASP855, PHE856, GLY857). These interactions are now explicitly highlighted in the updated figures.

Specifically, revised Figure 3 provides an improved 2D representation of the binding pocket, where ligand positioning relative to these key residues is clearly visible. In addition, a new Figure 4 has been included to explicitly present the 3D interaction profiles of all compounds with the critical hinge residues (GLN791, MET793) and other essential structural elements.

Reviewer #2: There is a jump from computational prediction to the implications of therapeutic effectiveness.

R1. There is no benchmarking with established EGFRwt inhibitors; therefore, the binding affinity and dynamics cannot be fully evaluated.

No comparison with known EGFR inhibitors.

A1. We sincerely appreciate your valuable comment regarding the importance of benchmarking against established EGFRwt inhibitors. As correctly noted, the present study is grounded in a drug repurposing strategy, transitioning from c-MET targeted compounds to EGFR, specifically the wild-type receptor. The investigated molecules were selected from a library of Food and Drug Administration (FDA)-approved kinase inhibitors.

Given the high degree of structural conservation within the kinase family, our objective was to explore the potential of these approved compounds to interact effectively with EGFRwt through rigorous in silico simulations, thereby identifying promising candidates in a time- and cost-efficient manner. We fully acknowledge the importance of benchmarking ; however, the primary scope of this work is predictive screening.

Importantly, we emphasize the critical role of subsequent bench experimental validation to confirm these findings. In this regard, we have added a dedicated paragraph in the revised manuscript (page 16, lines 495–501) highlighting the necessity of experimental benchmarking against established EGFR inhibitors to substantiate and extend the computational results.

Attachment Submitted filename: respond to reviewers.docx

# Peer review: Decision Letter 1

**DOI:** 10.1371/journal.pone.0350847.r003

**Authors:** Wagdy Eldehna

8 May 2026

PONE-D-26-01840R1

Identifying Potential Inhibitors for Wild-Type EGFR Tyrosine Kinase through a Cross-Talk Pathway Strategy and an In-Silico Drug Repurposing Method

PLOS One

Dear Dr. Abdelmalek,

Thank you for submitting your manuscript to PLOS ONE. After careful consideration, we feel that it has merit but does not fully meet PLOS ONE’s publication criteria as it currently stands. Therefore, we invite you to submit a revised version of the manuscript that addresses the points raised during the review process.

Please submit your revised manuscript by Jun 22 2026 11:59PM. If you will need more time than this to complete your revisions, please reply to this message or contact the journal office at plosone@plos.org. When you're ready to submit your revision, log on to https://www.editorialmanager.com/pone/ and select the 'Submissions Needing Revision' folder to locate your manuscript file.

Please include the following items when submitting your revised manuscript:

- A letter that responds to each point raised by the academic editor and reviewer(s). You should upload this letter as a separate file labeled 'Response to Reviewers'.
- A marked-up copy of your manuscript that highlights changes made to the original version. You should upload this as a separate file labeled 'Revised Manuscript with Track Changes'.
- An unmarked version of your revised paper without tracked changes. You should upload this as a separate file labeled 'Manuscript'.

If you would like to make changes to your financial disclosure, please include your updated statement in your cover letter. Guidelines for resubmitting your figure files are available below the reviewer comments at the end of this letter.

If applicable, we recommend that you deposit your laboratory protocols in protocols.io to enhance the reproducibility of your results. Protocols.io assigns your protocol its own identifier (DOI) so that it can be cited independently in the future. For instructions see: https://journals.plos.org/plosone/s/submission-guidelines#loc-laboratory-protocols. Additionally, PLOS ONE offers an option for publishing peer-reviewed Lab Protocol articles, which describe protocols hosted on protocols.io. Read more information on sharing protocols at https://plos.org/protocols?utm_medium=editorial-email&utm_source=authorletters&utm_campaign=protocols.

As the corresponding author, your ORCID iD is verified in the submission system and will appear in the published article. PLOS supports the use of ORCID, and we encourage all coauthors to register for an ORCID iD and use it as well. Please encourage your coauthors to verify their ORCID iD within the submission system before final acceptance, as unverified ORCID iDs will not appear in the published article. *Only*  the individual author can complete the verification step; PLOS staff *cannot*  verify ORCID iDs on behalf of authors.

We look forward to receiving your revised manuscript.

Kind regards,

Wagdy M. Eldehna, Ph.d

Academic Editor

PLOS One

Journal Requirements:

1. If the reviewer comments include a recommendation to cite specific previously published works, please review and evaluate these publications to determine whether they are relevant and should be cited. There is no requirement to cite these works unless the editor has indicated otherwise.

2. Please review your reference list to ensure that it is complete and correct. If you have cited papers that have been retracted, please include the rationale for doing so in the manuscript text, or remove these references and replace them with relevant current references. Any changes to the reference list should be mentioned in the rebuttal letter that accompanies your revised manuscript. If you need to cite a retracted article, indicate the article’s retracted status in the References list and also include a citation and full reference for the retraction notice.

[Note: HTML markup is below. Please do not edit.]

Reviewers' comments:

Reviewer's Responses to Questions

**Comments to the Author**

1. If the authors have adequately addressed your comments raised in a previous round of review and you feel that this manuscript is now acceptable for publication, you may indicate that here to bypass the “Comments to the Author” section, enter your conflict of interest statement in the “Confidential to Editor” section, and submit your "Accept" recommendation.

Reviewer #1: All comments have been addressed

Reviewer #2: All comments have been addressed

Reviewer #3: (No Response)

**********

2. Is the manuscript technically sound, and do the data support the conclusions?

The manuscript must describe a technically sound piece of scientific research with data that supports the conclusions. Experiments must have been conducted rigorously, with appropriate controls, replication, and sample sizes. The conclusions must be drawn appropriately based on the data presented.

Reviewer #1: Yes

Reviewer #2: Yes

Reviewer #3: Partly

**********

3. Has the statistical analysis been performed appropriately and rigorously?

Reviewer #1: N/A

Reviewer #2: N/A

Reviewer #3: I Don't Know

**********

4. Have the authors made all data underlying the findings in their manuscript fully available?

The PLOS Data policy requires authors to make all data underlying the findings described in their manuscript fully available without restriction, with rare exception (please refer to the Data Availability Statement in the manuscript PDF file). The data should be provided as part of the manuscript or its supporting information, or deposited to a public repository. For example, in addition to summary statistics, the data points behind means, medians and variance measures should be available. If there are restrictions on publicly sharing data—e.g. participant privacy or use of data from a third party—those must be specified.

Reviewer #1: Yes

Reviewer #2: Yes

Reviewer #3: No

**********

5. Is the manuscript presented in an intelligible fashion and written in standard English?

PLOS ONE does not copyedit accepted manuscripts, so the language in submitted articles must be clear, correct, and unambiguous. Any typographical or grammatical errors should be corrected at revision, so please note any specific errors here.

Reviewer #1: Yes

Reviewer #2: Yes

Reviewer #3: No

**********

6. Review Comments to the Author

Please use the space provided to explain your answers to the questions above. You may also include additional comments for the author, including concerns about dual publication, research ethics, or publication ethics. (Please upload your review as an attachment if it exceeds 20,000 characters)

Reviewer #1: Dear Authors,

I am pleased to inform you that your manuscript entitled “Identifying Potential Inhibitors for Wild-Type EGFR Tyrosine Kinase through a Cross Talk Pathway Strategy and an In-Silico Drug Repurposing Method” has been accepted for publication.

Reviewer #2: (No Response)

Reviewer #3: The revised manuscript by Abdelmalek et al. represents a meaningful response to the concerns raised in the first review cycle. The authors have addressed the two most critical methodological weaknesses: the structural justification for PDB ID 4I23 is now clearly articulated in the Materials and Methods section, and the binding mode analysis has been significantly strengthened through re-execution of induced-fit docking at 100 runs, with the revised figures now explicitly demonstrating interactions with the pharmacologically essential hinge residues GLN791 and MET793, the gatekeeper THR790, and the DFG motif. The addition of Figure 4 providing dual 2D/3D visualization of the binding pocket represents a genuine improvement in interpretive clarity. The computational pipeline, combining DrugRepoBank screening, dual-software docking validation, 100 ns GROMACS molecular dynamics, PCA/FEL analysis, and MM/PBSA free energy decomposition, is methodologically appropriate for a repurposing study of this scope, and Foretinib's emergence as the lead candidate is consistently and convergently supported across all metrics. The drug repurposing rationale exploiting MET/EGFR pathway crosstalk is scientifically well-grounded and clinically relevant given the underserved EGFRwt patient population.

However, prior to final acceptance, three minor but necessary corrections must be addressed:

The AutoDock Tools version placeholder ("version X.X") at line 558 must be completed.

The textual claim that D4 exhibits the highest ligand efficiency should be reconciled with Table 2, which shows D1 and D5 at 0.40 versus D4 at 0.37

The ranking of interaction strength stated in the text at lines 280–281 (D4 > D2 > D1 > D5 > D3) is based on qualitative structural engagement rather than raw docking affinity scores, which follow a different order in Table 2 (D4 > D3 > D1 > D2 > D5). While this distinction is scientifically defensible, it is never explicitly acknowledged in the text and is likely to confuse readers. I believe that a brief clarifying sentence noting that the two rankings reflect different evaluation criteria, quantitative binding affinity versus qualitative interaction richness, would resolve this ambiguity.

Additionally, despite the authors' acknowledgement of language editing support, several typographical errors persist throughout the manuscript(e.g., "describved," "strenghted," "trmed," "Simualtions," "rigourous," "biddable" and "leftt") and should be corrected before publishing the paper.

**********

7. PLOS authors have the option to publish the peer review history of their article (what does this mean?). If published, this will include your full peer review and any attached files.

If you choose “no”, your identity will remain anonymous but your review may still be made public.

**Do you want your identity to be public for this peer review?**  For information about this choice, including consent withdrawal, please see our Privacy Policy.

Reviewer #1: **Yes:**  Abdulrahman M. Saleh

Reviewer #2: **Yes:**  Mahmoud Rashed

Reviewer #3: No

**********

[NOTE: If reviewer comments were submitted as an attachment file, they will be attached to this email and accessible via the submission site. Please log into your account, locate the manuscript record, and check for the action link "View Attachments". If this link does not appear, there are no attachment files.]

To ensure your figures meet our technical requirements, please review our figure guidelines: https://journals.plos.org/plosone/s/figures

You may also use PLOS’s free figure tool, NAAS, to help you prepare publication quality figures: https://journals.plos.org/plosone/s/figures#loc-tools-for-figure-preparation.

NAAS will assess whether your figures meet our technical requirements by comparing each figure against our figure specifications.

# Author comment: Author response to Decision Letter 2

**DOI:** 10.1371/journal.pone.0350847.r004

12 May 2026

Response to Decision Letter and Reviewer Comments

(Manuscript number: PONE-D-26-01840R1)

Manuscript title: Identifying Potential Inhibitors for Wild-Type EGFR Tyrosine Kinase through a Cross-Talk Pathway Strategy and an In-Silico Drug Repurposing Method

Dear Respected Professor Wagdy M. Eldehna,

Academic Editor

Journal of Plos one

Mai, 12, 2026

We greatly appreciate your kind appreciation of the revision made to make the paper as its best scientific level. In this minor revision, we have taken also into consideration all Highly Respectful suggestions and comments raised by the reviewer.

We would therefore be pleased to resubmit a revised version of our improved manuscript which addresses all concerns and requests. Those changes are highlighted within the manuscript in yellow.

Please find below our response, for every single point raised by the reviewer. All page numbers refer to the final revised manuscript file with track changes in yellow.

We would be glad if you could consider our revised manuscript for possible publication in the Journal of Plos one

Once again, thank you indeed for your kind consideration.

On behalf of all co-authors,

Corresponding author:

Dr. Dorra Abdelmalek

Laboratory of Molecular and Cellular Screening Processes,

Centre of Biotechnology of Sfax,

University of Sfax, 3041 Sfax, Tunisia

Email: dorra.abdelmalek@cbs.rnrt.tn

Reviewer #3: The revised manuscript by Abdelmalek et al. represents a meaningful response to the concerns raised in the first review cycle. The authors have addressed the two most critical methodological weaknesses: the structural justification for PDB ID 4I23 is now clearly articulated in the Materials and Methods section, and the binding mode analysis has been significantly strengthened through re-execution of induced-fit docking at 100 runs, with the revised figures now explicitly demonstrating interactions with the pharmacologically essential hinge residues GLN791 and MET793, the gatekeeper THR790, and the DFG motif. The addition of Figure 4 providing dual 2D/3D visualization of the binding pocket represents a genuine improvement in interpretive clarity. The computational pipeline, combining DrugRepoBank screening, dual-software docking validation, 100 ns GROMACS molecular dynamics, PCA/FEL analysis, and MM/PBSA free energy decomposition, is methodologically appropriate for a repurposing study of this scope, and Foretinib's emergence as the lead candidate is consistently and convergently supported across all metrics. The drug repurposing rationale exploiting MET/EGFR pathway crosstalk is scientifically well-grounded and clinically relevant given the underserved EGFRwt patient population.

However, prior to final acceptance, three minor but necessary corrections must be addressed:

R1. The AutoDock Tools version placeholder ("version X.X") at line 558 must be completed.

A1. Yes, indeed it is Autodock tools -1.5.6rc3. we corrected this in the revised manuscript at lines 186, 554 and 559.

R2. The textual claim that D4 exhibits the highest ligand efficiency should be reconciled with Table 2, which shows D1 and D5 at 0.40 versus D4 at 0.37 The ranking of interaction strength stated in the text at lines 280–281 (D4 > D2 > D1 > D5 > D3) is based on qualitative structural engagement rather than raw docking affinity scores, which follow a different order in Table 2 (D4 > D3 > D1 > D2 > D5). While this distinction is scientifically defensible, it is never explicitly acknowledged in the text and is likely to confuse readers. I believe that a brief clarifying sentence noting that the two rankings reflect different evaluation criteria, quantitative binding affinity versus qualitative interaction richness, would resolve this ambiguity.

A2. Thank you for this interesting remark and for the suggestion to resolve the ambiguity. For that we corrected that D ligand efficiency score is among the best quantitative score, lines 221 and 222.

And for the qualitative scoring order we changed the text as follow: “Overall, based on the qualitative interaction richness, we note that the structural insights from Figure 4 reinforce the trends observed in Figure 3, supporting the ranking of interaction strength and binding engagement as D4 > D2 > D1 > D5 > D3. » lines 279-281 in the revised manuscript.

R3. Additionally, despite the authors' acknowledgement of language editing support, several typographical errors persist throughout the manuscript (e.g., "describved," "strenghted," "trmed," "Simualtions," "rigourous," "biddable" and "leftt") and should be corrected before publishing the paper.

A3. Thank you for taking care of these typing mistakes. As per recommended all these typo errors has been carefully corrected and highlighted in yellow.

Attachment Submitted filename: Response to Reviewers.docx

# Peer review: Decision Letter 2

**DOI:** 10.1371/journal.pone.0350847.r005

**Authors:** Wagdy Eldehna

20 May 2026

Identifying Potential Inhibitors for Wild-Type EGFR Tyrosine Kinase through a Cross-Talk Pathway Strategy and an In-Silico Drug Repurposing Method

PONE-D-26-01840R2

Dear Dr. Abdelmalek,

We’re pleased to inform you that your manuscript has been judged scientifically suitable for publication and will be formally accepted for publication once it meets all outstanding technical requirements.

Within one week, you’ll receive an e-mail detailing the required amendments. When these have been addressed, you’ll receive a formal acceptance letter and your manuscript will be scheduled for publication.

An invoice will be generated when your article is formally accepted. Please note, if your institution has a publishing partnership with PLOS and your article meets the relevant criteria, all or part of your publication costs will be covered. Please make sure your user information is up-to-date by logging into Editorial Manager at Editorial Manager® and clicking the ‘Update My Information' link at the top of the page. For questions related to billing, please contact billing support.

If your institution or institutions have a press office, please notify them about your upcoming paper to help maximize its impact. If they’ll be preparing press materials, please inform our press team as soon as possible -- no later than 48 hours after receiving the formal acceptance. Your manuscript will remain under strict press embargo until 2 pm Eastern Time on the date of publication. For more information, please contact onepress@plos.org.

Kind regards,

Wagdy M. Eldehna, Ph.d

Academic Editor

PLOS One

Additional Editor Comments (optional):

Reviewers' comments:

Reviewer's Responses to Questions

**Comments to the Author**

1. If the authors have adequately addressed your comments raised in a previous round of review and you feel that this manuscript is now acceptable for publication, you may indicate that here to bypass the “Comments to the Author” section, enter your conflict of interest statement in the “Confidential to Editor” section, and submit your "Accept" recommendation.

Reviewer #3: All comments have been addressed

**********

2. Is the manuscript technically sound, and do the data support the conclusions?

The manuscript must describe a technically sound piece of scientific research with data that supports the conclusions. Experiments must have been conducted rigorously, with appropriate controls, replication, and sample sizes. The conclusions must be drawn appropriately based on the data presented.

Reviewer #3: Yes

**********

3. Has the statistical analysis been performed appropriately and rigorously?

Reviewer #3: I Don't Know

**********

4. Have the authors made all data underlying the findings in their manuscript fully available?

The PLOS Data policy requires authors to make all data underlying the findings described in their manuscript fully available without restriction, with rare exception (please refer to the Data Availability Statement in the manuscript PDF file). The data should be provided as part of the manuscript or its supporting information, or deposited to a public repository. For example, in addition to summary statistics, the data points behind means, medians and variance measures should be available. If there are restrictions on publicly sharing data—e.g. participant privacy or use of data from a third party—those must be specified.

Reviewer #3: No

**********

5. Is the manuscript presented in an intelligible fashion and written in standard English?

PLOS ONE does not copyedit accepted manuscripts, so the language in submitted articles must be clear, correct, and unambiguous. Any typographical or grammatical errors should be corrected at revision, so please note any specific errors here.

Reviewer #3: Yes

**********

6. Review Comments to the Author

Please use the space provided to explain your answers to the questions above. You may also include additional comments for the author, including concerns about dual publication, research ethics, or publication ethics. (Please upload your review as an attachment if it exceeds 20,000 characters)

Reviewer #3: (No Response)

**********

7. PLOS authors have the option to publish the peer review history of their article (what does this mean?). If published, this will include your full peer review and any attached files.

If you choose “no”, your identity will remain anonymous but your review may still be made public.

**Do you want your identity to be public for this peer review?**  For information about this choice, including consent withdrawal, please see our Privacy Policy.

Reviewer #3: No

**********

# Editor report: Acceptance letter

**DOI:** 10.1371/journal.pone.0350847.r006

**Authors:** Wagdy Eldehna

PONE-D-26-01840R2

PLOS One

Dear Dr. Abdelmalek,

I'm pleased to inform you that your manuscript has been deemed suitable for publication in PLOS One. Congratulations! Your manuscript is now being handed over to our production team.

At this stage, our production department will prepare your paper for publication. This includes ensuring the following:

* All references, tables, and figures are properly cited

* All relevant supporting information is included in the manuscript submission,

* There are no issues that prevent the paper from being properly typeset

You will receive further instructions from the production team, including instructions on how to review your proof when it is ready. Please keep in mind that we are working through a large volume of accepted articles, so please give us a few days to review your paper and let you know the next and final steps.

Lastly, if your institution or institutions have a press office, please let them know about your upcoming paper now to help maximize its impact. If they'll be preparing press materials, please inform our press team within the next 48 hours. Your manuscript will remain under strict press embargo until 2 pm Eastern Time on the date of publication. For more information, please contact onepress@plos.org.

You will receive an invoice from PLOS for your publication fee after your manuscript has reached the completed accept phase. If you receive an email requesting payment before acceptance or for any other service, this may be a phishing scheme. Learn how to identify phishing emails and protect your accounts at https://explore.plos.org/phishing.

If we can help with anything else, please email us at customercare@plos.org.

Thank you for submitting your work to PLOS ONE and supporting open access.

Kind regards,

PLOS ONE Editorial Office Staff

on behalf of

Dr. Wagdy M. Eldehna

Academic Editor

PLOS One
